# Background Parenchymal Enhancement in Breast MRI Correlates with Molecular Subtypes of Breast Cancer

**DOI:** 10.2174/0115734056347327250117073638

**Published:** 2025-03-17

**Authors:** Hongyu Liu, Xinyue Chen, Yanna Wang, Xiaoping Yang, Yuxingzi Chen

**Affiliations:** 1 Department of Radiology, Taizhou Central Hospital, Taizhou, China; 2 Department of Nuclear Medicine, Taizhou Central Hospital, Taizhou, China

**Keywords:** Breast cancer, Background parenchymal enhancement, Magnetic resonance imaging, Dynamic enhancement, Molecular subtype, Fibroglandular Tissue (FGT)

## Abstract

**Purpose:**

MRI could be considered as a non-destructive disease diagnosis procedure, this procedure does not allow directly molecular types of cancer. Herein, we aimed to evaluate the correlation of breast MRI background parenchymal enhancement (BPE) and fibroglandular tissue (FGT) with the molecular subtypes and immunohistochemical markers of breast cancer.

**Methods:**

This was a single-cross-sectional retrospective study.Fifty-six patients diagnosed with unilateral breast cancer who underwent breast MRI scans before needle biopsy or surgery were selected. The relationship between qualitative and quantitative BPE/FGT ratios and the expression of breast cancer molecular subtypes and immunohistochemical markers were evaluated in patients with breast cancer.

**Results:**

Quantitative BPE (BPE%) of luminal A and luminal B was significantly lower than that of triple-negative breast cancer. There was no significant difference in the qualitative BPE/FGT between the different breast cancer subtypes. The quantitative BPE (BPE%) of estrogen receptor (ER)-negative tumors was higher than that of the ER-positive tumors, and the expression of FGT%, BPE%, and other immunohistochemical markers (human epidermal growth factor receptor-2(HER-2), progesterone receptor (PR), and Ki-67) were not significantly different. The proportion of high BPE distribution in HER-2 positive tumors was higher than that in the HER-2 negative group; however, there was no significant difference in the expression of qualitative BPE/FGT and other immunohistochemical markers (ER, PR, and Ki-67).

**Conclusion:**

There were significant differences in the levels of BPE among the different molecular subtypes. Therefore, BPE may be a potential imaging biomarker for the diagnosis of the molecular subtypes of breast cancer.

## 1 INTRODUCTION

Breast cancer has surpassed lung cancer as the most common cancer globally, and its mortality rate remains high [1]. There is a high degree of heterogeneity, and the determination of molecular subtypes is important for selecting treatment methods and evaluating prognosis. However, the recent decision regarding breast cancer molecular subtypes is based on immunohistochemical methods of invasive tissue sampling. It is imperative to seek non-invasive tissue sampling, accurately assess the biological behavior of the tumor, clarify the staging, formulate an appropriate personalized diagnosis and treatment plan, and provide a reference method for evaluating prognosis.

Studies have found that the amount of Breast Fibroglandular Tissue (FGT) is related to the risk of breast cancer, and background parenchymal enhancement (BPE) is associated with the risk of breast cancer [2-4, 21, 22]. The correlation between BPE and the molecular subtypes of breast cancer remains controversial [5-7]. This study explored the relationship between BPE and FGT and the expression of breast cancer molecular subtypes and immunohistochemical markers to provide a prospective option for distinguishing molecular subtypes of breast cancer. This is also the innovation point of this study, through non-invasive methods, to evaluate the biological behavior of tumors, reduce unnecessary invasive examinations, and maintain the physical and mental health of patients.

## 2 MATERIAL AND METHODS

### 2.1 General Information

This was a single-cross-sectional retrospective study. We retrospectively analyzed the clinical and imaging procedure data of 56 patients with breast cancer with different molecular subtypes confirmed by surgery and pathology at Taizhou Central Hospital (Taizhou University Hospital) from October 2018 to December 2020. All patients underwent plain MRI and enhanced scanning before surgery.

### 2.2 The Inclusion Criteria and the Exclusion Criteria

The inclusion criteria were as follows: (1) unilateral breast cancer; (2) breast MRI examination performed on days 7-14 between periods (menopausal patients did not have this requirement); (3) no diagnosis and treatment measures such as needle biopsy, surgery or radiotherapy, and chemotherapy performed before MRI examination; (4) breast surgery or needle biopsy performed within 2 weeks after MRI dynamic enhanced scan to obtain clear pathological and immunohistochemical results; (5) breast MRI performed on machines of the same model, with complete data; and (6) complete immunohistochemical data, including ER, PR, HER-2, and Ki-67 expression status.

The exclusion criteria were as follows: (1) a history of malignant tumors in other parts of the body; (2) pregnancy or breastfeeding within 6 months; (3) claustrophobia, contrast medium allergic reactions, and other examination contraindications; (4) administration of oral contraceptives, postmenopausal hormone therapy, or other hormone medications; (5) trauma in the breast area within one week before MRI examination; and (6) poor image quality.

### 2.3 Apparatus and Method

The United States General Electric Company Discovery MR750 3.0T superconducting magnetic resonance scanner and an 8-channel phased-array breast-dedicated coil were used. The examinee took the prone position, and the breasts on both sides hung naturally in the coil. The scanning sequence includes (1) conventional three-plane positioning and correction. (2) Axial T2WI-IDEAL scanning, repetition time (TR) 5284.0 ms, echo time (TE) 85.0 ms, layer thickness 4.0 mm, interval 1.0 mm, matrix 320 × 256, field-of-view (FOV) 32 cm × 32 cm, number of excitations (NEX) 1. (3) Axial T1WI scan, TR 420.0 ms, TE 6.4 ms, layer thickness 4.0 mm, interval 1.0 mm, matrix 320 × 256, FOV 32 cm × 32 cm, NEX 1. (4) Double breast sagittal T2WI fat suppression scan, TR 3500.0 ms, TE 85.0 ms, slice thickness 4.0 mm, interval 1.0 mm, matrix 288 × 224, FOV 20 cm × 20 cm, NEX 2. (5) Axial diffusion-weighted imaging (DWI) scan, TR 3600 ms, TE Minimum, layer thickness 4.0 mm, interval 1.0 mm, matrix 128 × 128, FOV 32 cm × 32 cm; b value is 0, 1000 s/mm2. (6) Axial dynamic enhanced scanning, using 3D-VIBRANT sequence, TR 4.2 ms, TE 2.1 ms, layer thickness 1.6 mm, no interval scanning, matrix 320 × 256, FOV 36 cm × 36 cm, NEX 1.

The mask was first scanned, after which the contrast agent gadopentetate meglumine (Gd-DTPA) was injected through the cubital vein at a flow rate of 2.0 ml/s and a dose of 0.1 mmol/kg and then followed by a 20-ml saline flush. Seven phases were continuously collected, and each phase took 58–60 s to acquire.

### 2.4 Image Analysis

#### 2.4.1 Qualitative Analysis of BPE and FGT

**Fig. (1):**
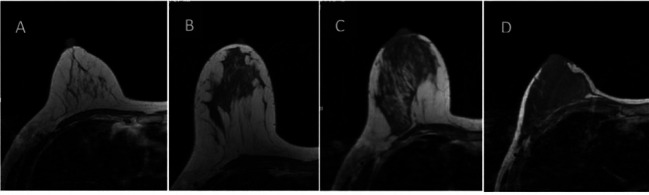
Classification of FGT on T1WI image of the breast. Almost completely fat (**A**), scattered in glandular tissue (**B**), mixed glandular tissue (**C**), and dense glandular tissue (**D**).

**Fig. (2):**
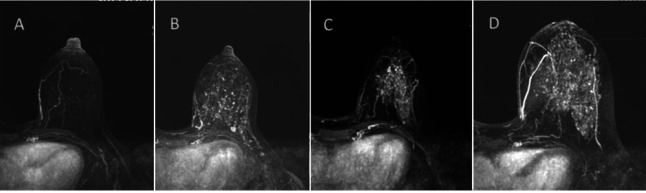
A qualitative classification of BPE on the early MIP image after enhancement. Little reinforcement (**A**), mild reinforcement (**B**), moderately strengthened (**C**), and significantly strengthened (**D**).

On the GE AW4.6 workstation, according to the 2013 BI-RADS standard, the BPE and FGT were qualitatively evaluated on the T1WI before enhancement and the early maximum intensity projection (MIP) image after dynamic enhancement [8]. FGT was classified into 4 categories: Level 1 is almost completely fat (gland content ≤ 25%), level 2 is scattered glandular tissue (glandular content 26%–50%), level 3 is mixed glandular tissue (glandular content) 51%–75%) and level 4 is dense gland tissue (glandular content > 75%). Furthermore, FGT is divided into low FGT (grade 1, 2) and high FGT (grade 3, 4) (Fig. **1**). Similarly, BPE is divided into 4 categories: minimal enhancement (≤ 25% gland enhancement), mild enhancement (26%–50% gland enhancement), moderate enhancement (51%–75% gland enhancement), and significant enhancement (> 75% gland enhancement) (Fig. **2**). We classified minimal-mild enhancement as low BPE and moderate-significant enhancement as high BPE. Afterward, we selected the contralateral (no tumor) breast for evaluation to avoid tumors in the affected breast causing increased blood supply or increased permeability and carried out BPE examinations.

#### 2.4.2 Quantitative Analysis of BPE and FGT

**Fig. (3):**
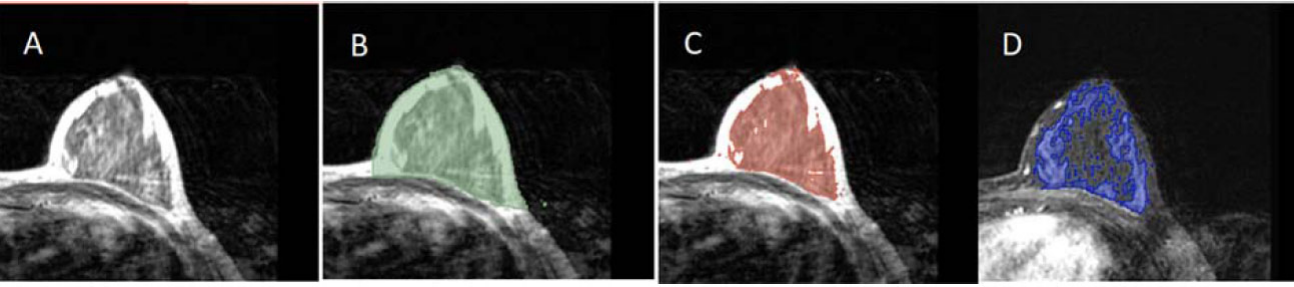
Quantitative segmentation of breast BPE. T1WI original image (**A**), segmentation of breast tissue from the chest wall and background (green; **B**); FGT segmentation on T1WI image (red; **C**); BPE segmentation on the enhanced early (phase 2) image (blue; **D**).

**Fig. (4):**
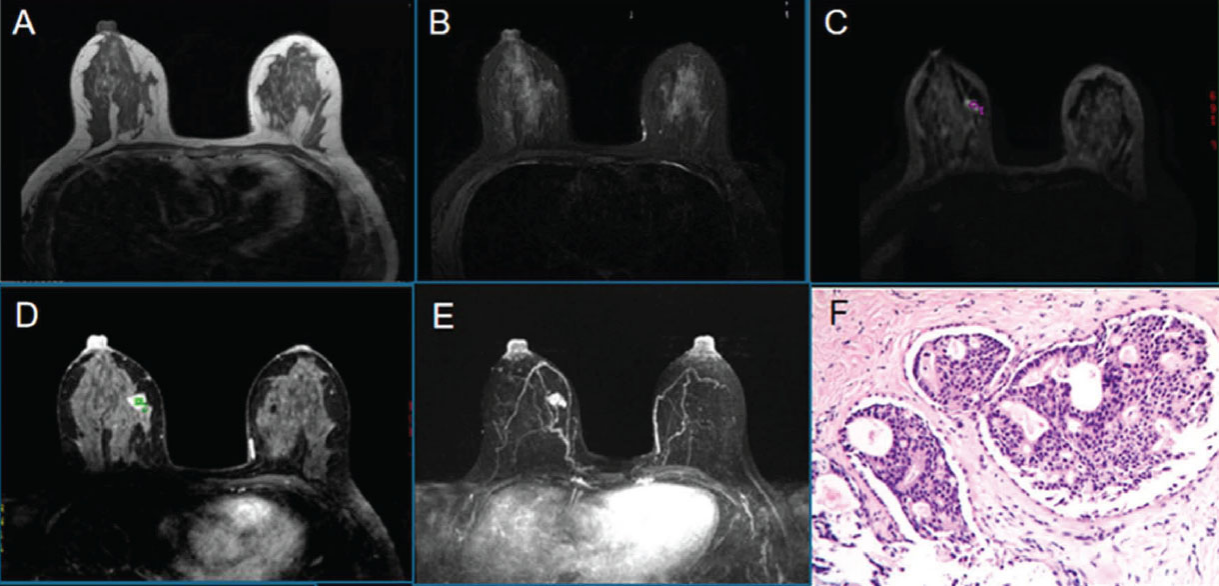
Female, 44 years old, right breast duct carcinoma *in situ*, Luminal A, FGT is a mixed gland type. T1WI (**A**), T2WI-fs (**B**), DWI (**C**), the early lesions on dynamic enhanced scan (**D**), MIP (**E**), Pathological microscopy (HE×100) (**F**).

**Fig. (5):**
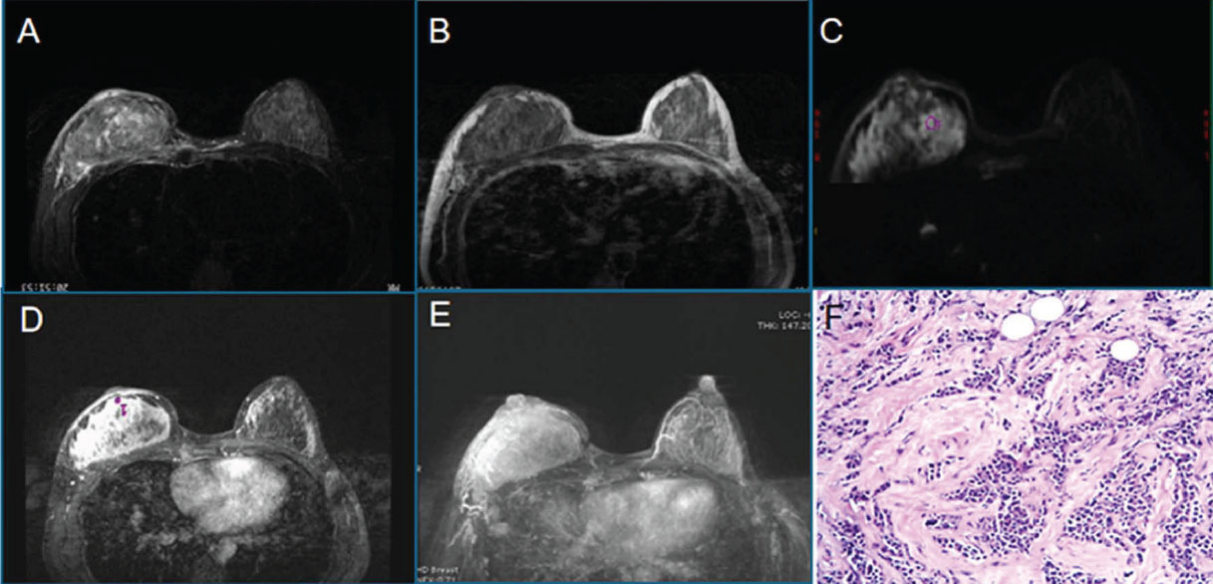
Female, 37 years old, with invasive ductal carcinoma (NST) of the right breast, Luminal B, FGT is a dense gland type. T2WI-fs (**A**), T1WI (**B**), DWI (**C**), dynamic enhancement scanning (**D**), MIP (**E**), Pathological microscopy(HE×100) (**F**).

**Fig. (6):**
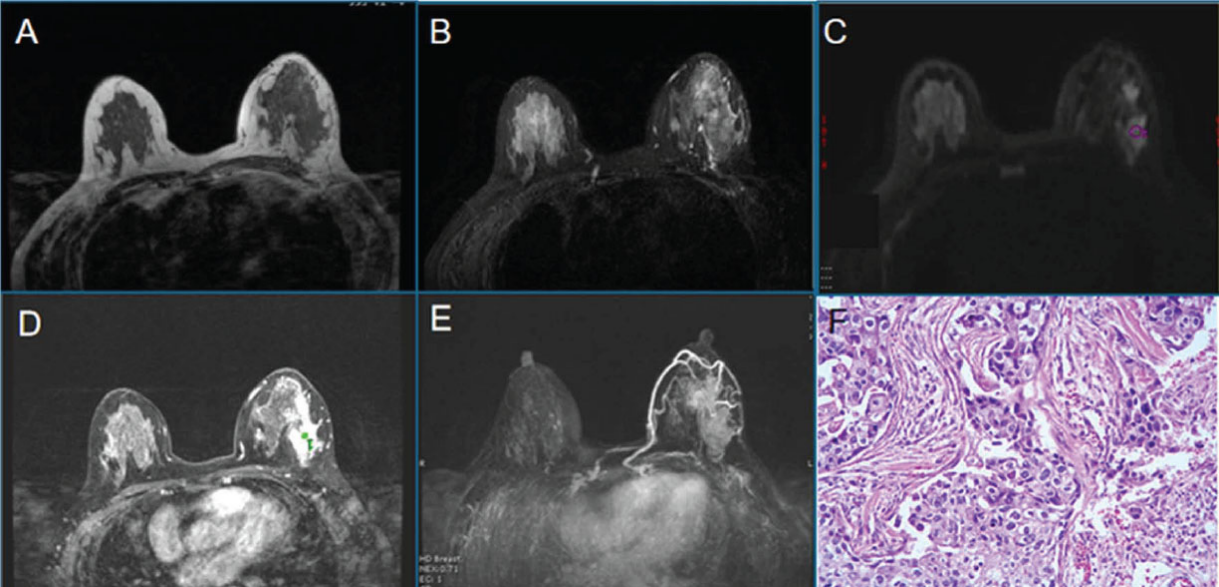
Female, 49 years old, an NST with HER-2 overexpression. FGT is a mixed gland type. T1WI (**A**), T2WI-fs (**B**), DWI (**C**), the dynamic enhancement scan (**D**), MIP (**E**), the pathological microscope. Mass(HE×100).

**Fig. (7):**
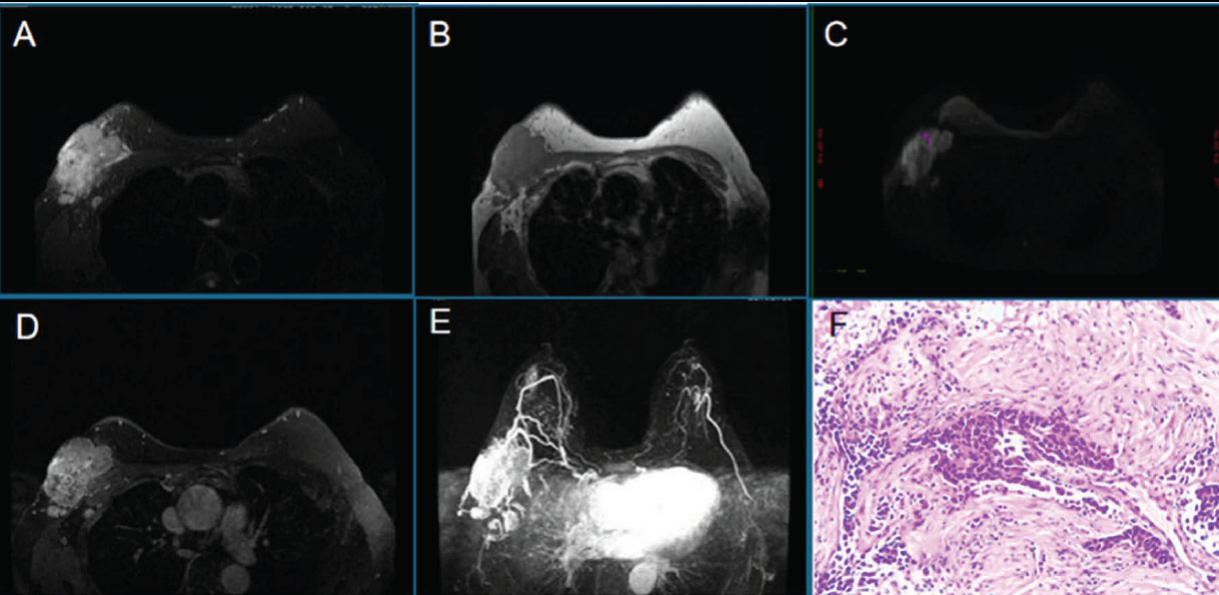
Female, 51 years old, with invasive ductal carcinoma.FGT is scattered glandular type. Right breast cancer (NST), triple-negative. T2WI-fs (**A**), T1WI (**B**), DWI (**C**), the early lesions on dynamic enhanced scan (**D**), MIP (**E**), pathological microscopy(HE×100).

Segmentation of breast and fibroglandular tissues on MRI images was based on semi-automatic segmentation [9-12]. Each patient's MRI examination data were imported into the software 3D slicer 4.11, semi-automatically segmenting the uninfected breast on the conventional T1WI image before enhancement, manually drawing a large area containing all breast parenchyma on each axial image and manually segmenting the entire breast edge. The breast was segmented from the image background and chest wall, from which the absolute total volume of the breast was estimated. The threshold level method was used to separate the breast glands from the fat tissues automatically. The FGT percentage was calculated as follows: FGT% = (FGT volume/volume of the entire breast) × 100%. In the second phase of the dynamic enhanced scan image, the BPE value was extracted from the enhanced voxels inside the breast tissue: BPE% = (BPE volume/FGT volume) × 100% (Fig. **3**). In addition, the representative breast carcinoma cases were validated by histomorphological and pathological detection as follows: a right breast duct carcinoma (no special type, NST) *in situ* with luminal A (Fig. **4**), FGT is a mixed gland type, immunohistochemical results: ER (+), PR (+), HER-2 (-), Ki-67 < 5% (F), and quantitative FGT and BPE of the left breast split, FGT% = 66.93%, BPE% = 27.06%.An NST of the right breast with luminal B (Fig. **5**), FGT is a dense gland type, immunohistochemical results: ER (+), PR (-), HER-2 (-), Ki-67 = 40%, and quantitative segmentation of FGT and BPE on the left breast, FGT% = 76.84%, BPE% = 80.82%.An NST with human epidermal growth factor receptor-2 (HER-2) overexpression (Fig. **6**), FGT is a mixed gland type, immunohistochemical results: ER (-), PR (-), HER-2 (3+), Ki-67 = 60% (F), and right breast FGT and BPE quantitative segmentation, FGT% = 46.35%, BPE% = 35.54%. An NST with triple-negative breast carcinoma (Fig. **7**).FGT is scattered glandular type, immunohistochemical results: ER (-), PR (-), HER-2 (-), Ki-67 = 40% (F), and quantitative segmentation of FGT and BPE of the right breast, FGT% = 40.51%, BPE% = 36.72%.

### 2.5 Pathological and Immunohistochemical Detection and Analysis

Paraffin-embedded sections that met the inclusion and exclusion criteria were subjected to hematoxylin and eosin staining. Immunohistochemistry (IHC) was used to assess estrogen receptor (ER), progesterone receptor (PR), HER-2, and Ki-67. The positive criteria for ER and PR were based on the 2010 American Society of Clinical Oncology/College of American Pathologists guideline recommendations for immunohistochemical testing of estrogen and progesterone receptors in breast cancer (unabridged version). HER-2-positive criteria were based on the 2007 (ASCO) guidelines for HER2 testing. Ki-67 was used immunohistochemically to calculate the percentage of positive cells in the densest area of the cells in the field under a high-power microscope, based on the expert consensus of the St. Gallen International Expert Consensus on the Primary Therapy of Early Breast Cancer 2013 [13-18].

According to the principle of double blindness, pathological specimens were evaluated by pathologists with experience in breast pathological diagnosis. According to the immunohistochemistry results, the breast cancer patients were divided into 4 molecular subtypes: (1) Luminal A: PR/ER-positive, and high expression of PR (≥ 20%), negative for HER-2, low expression of Ki-67 (< 20%); (2) Luminal B: HER-2 Negative: ER-positive, HER-2 negative, and at least one of the following conditions: Ki-67 high expression (≥ 20%), PR negative or low expression (< 20%); HER-2 positive: ER-positive, HER-2 Positive, Ki-67 in any state, PR in any state; (3) HER-2 overexpression: HER-2 positive (non-luminal), HER-2 overexpression or proliferation, ER and PR negative; (4) Triple-negative: ER, PR, and HER-2 were all negative, and Ki-67 was expressed arbitrarily. In this study, 20% was used as the cutoff value, < 20% tumor cell nuclear staining was defined as low expression of Ki-67, and ≥ 20% tumor cell nuclear staining was defined as high-level expression.

### 2.6 Data Processing and Statistical Analysis

SPSS software (version 25.0; IBM Corp. Armonk, NY, USA) was used for the statistical analysis. Quantitative BPE and FGT were analyzed and compared in each group using an independent-sample t-test or Mann-Whitney U test. Quantitative comparison of BPE and FGT in different breast cancer molecular subtypes was performed using one-way analysis of variance (one-way ANOVA) or multiple sample non-parametric test (Kruskal-Wallis H), and pairwise comparisons between groups were performed using the Bonferroni-t method or non-parametric test. For qualitative analysis, the distribution of high- and low-grade BPE and high- and low-grade FGT in different molecular subtypes of breast cancer and the proportion of immunohistochemical markers were expressed as percentages or rates, and the chi-square test was used for comparisons. The above results were considered statistically significant at *p* < 0.05.

## 3 RESULTS

### 3.1 Clinical and Pathological Data

According to the inclusion criteria, 56 cases were selected for the evaluations. The patients were all of the female gender. The age varied between 3 and 67 years, with an average age of 49.14 ± 7.98 years. Microscopy evaluations showed 43 cases of invasive ductal carcinoma, 4 cases of ductal carcinoma *in situ*, 2 cases of papillary ductal carcinoma *in situ*, 1 case of cribriform breast carcinoma, and 1 case of mixed breast carcinoma(ductal carcinoma *in situ* accounted for 60%, and lobular carcinoma for 40%), 1 case of small cell neuroendocrine carcinoma and 1 case of Paget's disease of the breast. The clinical and pathological data of patients with breast cancer are shown in Table **1**.

### 3.2 Classification of BPE and FGT on MRI of Breast Cancer Patients

In 56 cases of breast cancer, low BPE accounted for 53.57% (30/56), of which minimal enhancement accounted for 14.28% (8/56), mild enhancement accounted for 39.29% (22/56), and high BPE accounted for 46.43% (26/56).In these cases, 26.79% (15/56) were moderately strengthened, and 19.64% (11/56) were significantly strengthened. Low FGT accounted for 50.00% (28/56), of which FGT1 accounted for 16.07% (9/56), FGT2 accounted for 33.93% (19/56), and high FGT accounted for 50.00% (28/56), of which FGT3 accounted for 37.50% (21 /56), FGT4 accounted for 12.50% (7/56), as shown in Table **2**.

### 3.3 Qualitative relationship between BPE, FGT, and Molecular Subtypes of Breast Cancer

**Fig. (8):**
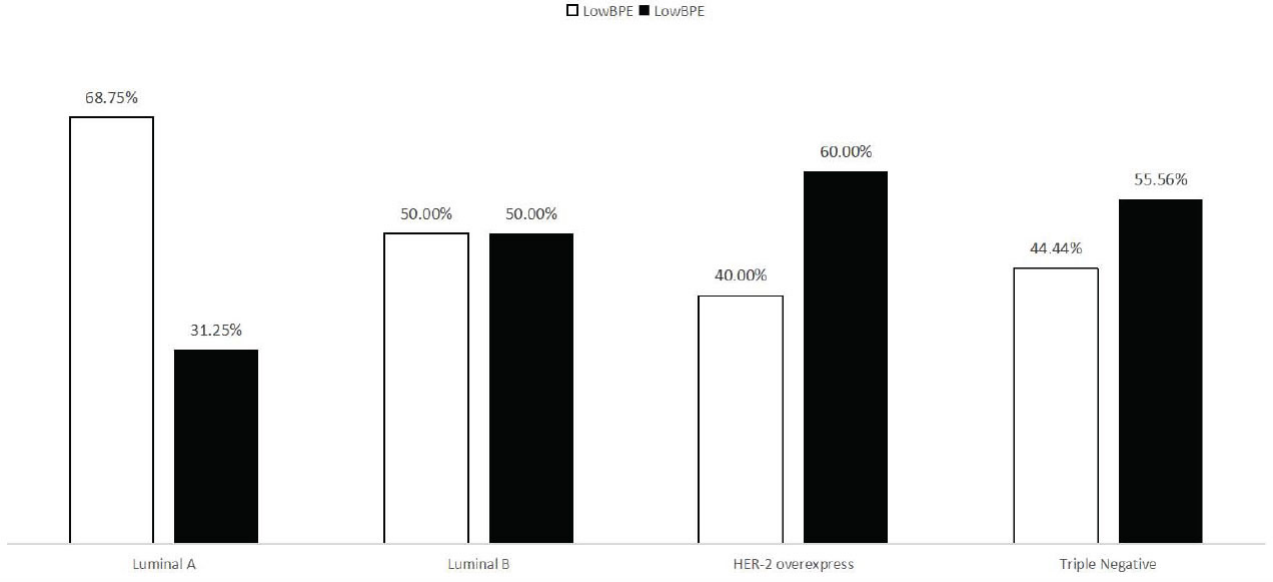
Comparison of qualitative BPE of different molecular subtypes of breast cancer cases.

**Fig. (9):**
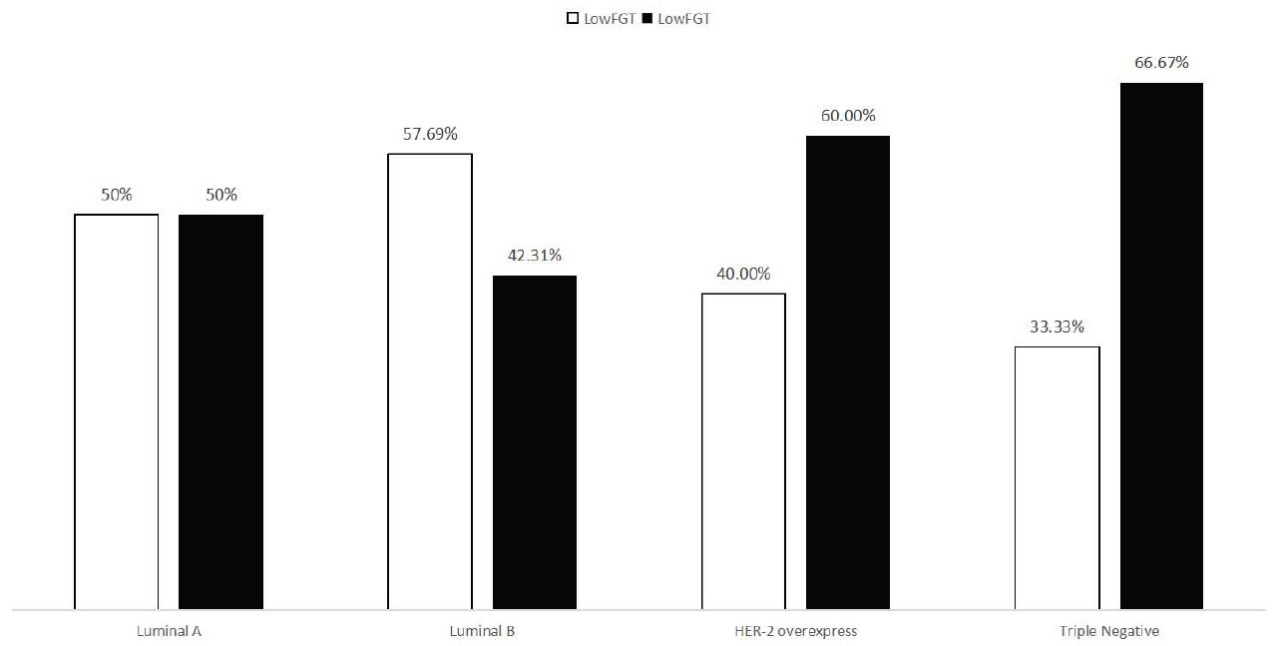
Comparison of qualitative FGT of different molecular subtypes of breast cancer cases.

Luminal A low-medium BPE accounted for 68.75% (11/16), high BPE accounted for 31.25% (5/16); Luminal B low-medium BPE accounted for 50.00% (13/26), high BPE accounted for 50.00% (13/26); HER-2 overexpression low-medium BPE accounted for 40.00% (2/5), high BPE accounted for 60.00% (3/5); Triple-negative low-medium BPE accounted for 44.44% (4/9), high BPE accounted for 55.56% (5/9). Chi-square test analysis revealed no significant difference in qualitative BEP among the different breast cancer subtypes (Fig. **8**). Luminal A type low and medium FGT accounted for 50.00% (8/16), high FGT accounted for 50.00% (8/16); Luminal B medium and low FGT accounted for 57.69% (15/26), high FGT accounted for 42.31% (11/26); HER-2 overexpression medium-low FGT accounted for 40.00% (2/5), high FGT accounted for 60.00% (3/5); Triple-negative medium-low FGT accounted for 33.33% (3/9), high FGT accounted for 66.67% (6/9)(Fig. **9**). The statistical analysis results showed no significant difference in qualitative FGT in the different breast cancer subtypes, as shown in Table **3**.

### 3.4 Quantification of 3.4 the relationship between BPE, FGT, and Breast Cancer Molecular Subtypes

Pairwise comparisons between groups showed that the BPE% of Luminal A and Luminal B were lower than those of triple-negative breast cancer, which was statistically significant (39.23 ± 15.53 *vs*. 57.75 ± 21.68, *p* = 0.01; 42.69 ± 14.82 *vs*. 57.75 ± 21.68, *p* = 0.02). However, there was no statistically significant difference in BPE% when compared to the other molecular subtype groups. There was no statistically significant difference in the FGT% between the different molecular subtypes of breast cancer (Table **4**).

### 3.5 Qualitative relationship between BPE and FGT and different IHC Markers

The proportion of high BPE distribution in HER-2 positive tumors was higher than that in the HER-2 negative group, and the difference between qualitative BPE in the HER-2 negative and positive groups was statistically significant. In addition, in the comparison of other groups, qualitative BPE showed no significant statistical difference in FGT (Table **5**).

### 3.6 Quantification of the relationship between BPE and FGT and different IHC Markers

The FGT% of the ER-negative group was higher than that of the ER-positive group, and the difference in FGT% between the ER-positive and ER-negative groups was statistically significant. There was no statistical difference between BPE% and FGT% among the groups (Table **6**).

## 4 DISCUSSION

In 2013, the fifth edition of MRI BI-RADS [13] defined and standardized the interpretation of BPE, believing that BPE is a physiological change and that BPE may represent changes in tissue microvascular and/or permeability regulated by endogenous hormones (mainly estrogen). It shows a dynamic process; the BPE of different women is different at different periods. BPE is common in breast enlargement tests; moderate-to-severe BPE or asymmetric or uneven BPE on MRI seriously interferes with the detection of lesions. However, other studies [8, 11, 14] showed that BPE is an essential cofactor in the growth, angiogenesis, metastasis, and immune response to breast tumors. The degree of enhancement is closely related to the risk of breast cancer and its therapeutic effect. BPE is highly correlated with female hormone levels and can potentially be used as an MRI marker of breast cancer risk. However, due to differences in experimental design, BPE assessment methods, inclusion criteria, MRI detection methods, contrast agent use, and other aspects, BPE and breast cancer molecular subtypes, hormone receptor and tumor marker expression levels, breast cancer risk, and prognosis are not consistent [14].

Breast MRI BPE refers to the enhancement of the normal FGT of the breast after injection of contrast medium. Exploring the value of BPE in breast disease diagnosis, breast cancer treatment, and prognosis assessment has gradually become a trending topic in current research, however, due to many differences in experimental design, BPE assessment methods, inclusion criteria, MRI detection methods, contrast agent use, BPE and breast cancer molecular subtypes, hormone receptors, tumor marker expression levels, breast cancer risk, and prognosis. Research results, such as predictions, have not been consistent [14].

This study explored the relationship between quantitative and qualitative BPE, breast cancer molecular subtypes, and immunohistochemical markers. The results showed that the BPE% of luminal A and luminal B patients was significantly lower than that of triple-negative breast cancer patients, and there was no significant difference in quantitative FGT% in different subtypes of breast cancer. Compared with luminal-type patients, patients with triple-negative breast cancer have a relatively poor prognosis and a higher recurrence rate, indicating that patients with high BPE may have a higher risk of breast cancer with poor prognoses [10]. However, we found that qualitative BPE cannot predict molecular subtypes like quantitative BPE [15, 16]. The reasons for this analysis may be because: (1) Since we used the same model of MRI, the parameter design was unified, and the inclusion standards were improved, which led to the collection of fewer samples, especially the smaller number of HER-2 overexpression groups. Thus, multi-center and extensive sample studies are needed to further verify the value of BPE in different molecular subtypes and clinicopathological biomarkers. (2) The qualitative evaluation had subjective observer bias; although the qualitative evaluation results were verified by quantitative evaluation, there is currently no standard BPE quantification method. Therefore, we used a semi-automatic method to quantitatively analyze BPE, despite the semi-quantitative method having certain degrees of subjective bias in the manual delineation process. In the future, a more objective, standardized, and repeatable automatic segmentation method will be required to accurately segment and quantify BPE. (3) There is no standard threshold range for quantitative BPE.

There have been few reports and opinions on the application of BPE in distinguishing molecular subtypes of breast cancer. Dilorenzo *et al*. [17] tested the relationship between BPE and molecular subtypes of breast cancer in 82 Italian women and found that mild BPE was more common in luminal B than in other levels of BPE. At the same time, moderate and significant BPE accounted for more in triple-negative breast cancer, which was consistent with our research results. Similarly, the clinicopathological and imaging characteristics of 2995 women undergoing mammography and breast MRI revealed that patients with triple-negative breast cancer had lower gland density [18], which was inconsistent with our research results. In terms of gland density, FGT, and BPE, women with low BPE were more than twice as likely to develop triple-negative breast cancer as women with high BPE. As mentioned earlier, there are many reasons for the inconsistency in experimental results, and this requires further improvement of the theoretical basis for the occurrence and development of BPE, unifying the methods and standards of BPE evaluation, and promoting the clinical transformation of BPE in breast cancer diagnosis and treatment.

There is also some controversy regarding the correlation between BPE and FGT and tumor markers [15, 19, 20]. Our results indicated that the FGT% of the ER-negative expression group was higher than that of the ER-positive group, while that of the HER-2-positive tumor was higher. The proportion of BPE distribution was higher than that of the HER-2-negative group, and there was no significant correlation between the expression levels of BPE, FGT, PR, and Ki-67, which is consistent with the results of previous studies [19]. HER-2 overexpression is an essential factor that affects the growth and metastasis of breast cancer. HER-2-positive breast cancer usually has a higher histological grade, higher metastasis rate, and poor prognosis [14]. Therefore, breast cancer patients with higher BPE levels may have a worse prognosis [21, 22].

## CONCLUSION

More preclinical and clinical trials are needed to study and explore the potential of BPE, an emerging tumor marker, to diagnose and treat breast cancer. Based on this study, BPE may be a potential tool for predicting luminal and triple-negative breast cancer diagnosis. It may be used as an imaging marker for prognostic evaluation and provide additional guidance for breast cancer treatment. In the future, we will continue to study the correlation between BPE level differences and tumor prognosis.

In light of this, we conclude that MRI is beneficial for distinguishing molecular subtypes of breast cancer. BPE by MRI differs between subtypes. The quantitative BPE of luminal A and B types was much lower than that of triple-negative cancer. BPE may be a useful diagnostic for detecting molecular subtypes of breast cancer. It can also improve the diagnosis and treatment accuracy of breast cancer patients prior to therapy, as well as predict cancer prognosis. BPE reduces invasive examinations of patients while maintaining their physical and emotional well-being.

## Figures and Tables

**Table 1:** Clinical and pathological data of breast cancer patients.

Clinical Information		n(%)
Pathological type	-	56 (100.00)
	Invasive ductal carcinoma(NST)	43 (76.78)
	Ductal carcinoma *in situ*	4 (7.14)
	Invasive lobular carcinoma	3 (5.35)
	Papillary ductal carcinoma *in situ*	2 (3.57)
	Cribriform breast cancer	1 (1.80)
	Mixed breast cancer	1 (1.80)
	Small cell neuroendocrine carcinoma (SNEC)	1 (1.80)
	Paget disease of the breast (PDB)	1 (1.80)
ER	Negative	14 (25.00)
	Positive	42 (75.00)
PR	Negative	25 (44.65)
	Positive	31 (55.35)
HER-2	Negative	41 (73.21)
	Positive	15 (26.79)
Ki-67	Low expression (<20%)	17 (30.36)
	High expression (≥20%)	39 (69.64)
Molecular subtype	Luminal A	16 (28.57)
	Luminal B	26 (46.43)
	HER-2 overexpression	5 (8.93)
	Triple-negative	9 (16.07)

**Table 2:** Classification of BPE and FGT in breast cancer patients case (%).

-	Low BPE		High BPE		Low FGT		High FGT	
	Rarely	Mild	Moderate	Significant	FGT1	FGT2	FGT3	FGT4
Luminal A	3 (18.75)	8 (50.00)	3 (18.75)	2 (12.50)	4 (25.00)	4 (25.00)	6 (37.50)	2 (12.50)
Luminal B	4 (15.38)	9 (34.62)	7 (26.92)	6 (23.08)	3 (11.54)	12 (46.15)	8 (30.77)	3 (11.54)
HER-2overexpression	0 (0.00)	2 (40.00)	2 (40.00)	1 (20.00)	1 (20.00)	1 (20.00)	2 (40.00)	1 (20.00)
Triple negative	1 (11.11)	3 (33.33)	4 (44.44)	1 (11.11)	1 (11.11)	2 (22.22)	5 (55.56)	1 (11.11)
ER negative	1 (7.14)	7 (50.00)	4 (28.57)	2 (14.29)	2 (14.29)	3 (21.42)	7 (50.00)	2 (14.29)
ER positive	7 (16.67)	15 (35.71)	11 (26.19)	9 (21.43)	7 (16.66)	16 (38.10)	16 (38.10)	3 (7.14)
PR negative	4 (16.00)	12 (48.00)	6 (24.00)	3 (12.00)	4 (16.00)	7 (28.00)	10 (40.00)	4 (16.00)
PR positive	5 (16.13)	9 (29.03)	9 (29.03)	8 (25.81)	5 (16.13)	12 (38.71)	11 (35.48)	3 (9.68)
HER-2 negative	8 (19.51)	18 (43.90)	8 (19.51)	7 (17.08)	7 (17.08)	15 (36.58)	14 (34.15)	5 (12.19)
HER-2 positive	1 (6.66)	3 (20.00)	7 (46.67)	4 (26.67)	2 (13.33)	4 (26.67)	7 (46.67)	2 (13.33)
Ki-67 Low expression	3 (17.65)	9 (52.94)	3 (17.65)	2 (11.76)	5 (29.41)	4 (23.53)	6 (35.29)	2 (11.77)
Ki-67 High expression	5 (12.82)	13 (33.33)	12 (30.77)	9 (23.08)	4 (10.26)	15 (38.46)	15 (38.46)	5 (12.82)

**Table 3:** Comparison of qualitative BPE and FGT of different molecular subtypes of breast cancer cases (%).

Group	BPE		*X* ^2^	*p*-value	FGT		*X* ^2^	*p*-value
	Low BPE (n=30)	HighBPE (n=26)			Low FGT(n=28)	High FGT(n=28)		
Luminal A	11 (68.75)	5 (31.25)	2.28	0.51	8 (50.00)	8 (50.00)	1.81	0.61
Luminal B	13 (50.00)	13 (50.00)			15 (57.69)	11 (42.31)		
HER-2 overexpression	2 (40.00)	3 (60.00)			2 (40.00)	3 (60.00)		
Triple negative	4 (44.44)	5 (55.56)			3 (33.33)	6 (66.67)		

**Table 4:** Comparison of quantitative BPE and FGT of different molecular subtypes of breast cancer.

-	Luminal A	Luminal B	HER-2 Overexpression	Triple-negative	F-value	*p*-value
BPE%	39.23±15.53	42.69±14.82	52.48±18.11	57.75±21.68	2.95	0.04
FGT%	38.87±16.60	38.01±12.40	41.35±15.70	47.13±22.80	0.79	0.50

**Table 5:** The relationship between qualitative BPE and FGT and different IHC markers case (%).

Group		BPE		*X* ^2^	*p*-value	FGT		*X* ^2^	*p*-value
		Low (n=30)	High (n=26)			Low(n=28)	High (n=28)		
ER	Negative	8 (57.14)	6 (42.86)	0.10	0.75	5 (35.71)	9 (64.29)	1.52	0.22
	Positive	22 (52.38)	20 (47.62)			23 (54.76)	19 (45.24)		
PR	Negative	16 (64.00)	9 (36.00)	1.98	0.16	11 (44.00)	14 (56.00)	0.65	0.42
	Positive	14 (45.16)	17 (54.84)			17 (54.84)	14 (45.16)		
HER-2	Negative	26 (63.41)	15 (36.59)	5.96	0.02	22 (53.66)	19 (46.34)	0.82	0.36
	Positive	4 (26.67)	11 (73.33)			6 (40.00)	9 (60.00)		
Ki-67	Low expression	12 (70.59)	5 (29.41)	2.84	0.09	9 (52.94)	8 (47.06)	0.08	0.77
	High expression	18 (46.15)	21 (53.85)			19 (48.72)	20 (51.28)		

**Table 6:** The relationship between quantitative BPE and FGT and different IHC markers.

-		BPE%	F-value	*p*-value	FGT%	F-value	*p*-value
ER	Negative	55.86±19.93	1.88	0.17	45.06±20.12	5.31	0.02
	Positive	41.37±15.01			38.33±13.72		
PR	Negative	46.73±19.47	1.91	0.17	42.09±17.20	1.05	0.31
	Positive	43.60±15.66			38.34±14.34		
HER-2	Negative	44.38±17.61	0.03	0.86	39.55±16.60	1.42	0.23
	Positive	46.67±17.19			41.29±13.11		
Ki-67	Low expression	39.56±15.10	1.21	0.27	38.73±15.56	0.01	0.93
	High expression	47.36±17.94			40.58±15.80		

## Data Availability

The raw data supporting the conclusions of this article will be made available by the corresponding author [Y.C] without undue reservation.

## LIST OF ABBREVIATIONS

BPE: Background Parenchymal Enhancement
FGT: Fibroglandular Tissue
PR: Progesterone Receptor

## STANDARDS OF REPORTING

STROBE guidelines were followed.

## References

[1] Siegel R.L., Miller K.D., Fuchs H.E., Jemal A. (2021). Cancer statistics, 2021.. CA Cancer J. Clin..

[2] Feng Y., Spezia M., Huang S., Yuan C., Zeng Z., Zhang L., Ji X., Liu W., Huang B., Luo W., Liu B., Lei Y., Du S., Vuppalapati A., Luu H.H., Haydon R.C., He T.C., Ren G. (2018). Breast cancer development and progression: Risk factors, cancer stem cells, signaling pathways, genomics, and molecular pathogenesis.. Genes Dis..

[3] Russnes H.G., Lingjærde O.C., Børresen-Dale A.L., Caldas C. (2017). Breast cancer molecular stratification.. Am. J. Pathol..

[4] Jung Y, Jeong SK, Kang DK, Moon Y, Kim TH (2018). Quantitative analysis of background parenchymal enhancement in whole breast on MRI: Influence of menstrual cycle and comparison with a qualitative analysis.. Eur. J. Radiol..

[5] Johansson ALV, Trewin CB, Fredriksson I, Reinertsen KV, Russnes H, Ursin G (2021). In modern times, how important are breast cancer stage, grade and receptor subtype for survival: A population-based cohort study.. Breast Cancer Res..

[6] Arasu V.A., Miglioretti D.L., Sprague B.L., Alsheik N.H., Buist D.S.M., Henderson L.M., Herschorn S.D., Lee J.M., Onega T., Rauscher G.H., Wernli K.J., Lehman C.D., Kerlikowske K. (2019). Population-based assessment of the association between magnetic resonance imaging background parenchymal enhancement and future primary breast cancer risk.. J. Clin. Oncol..

[7] Kim J.Y., Kim S.H., Kim Y.J., Kang B.J., An Y.Y., Lee A.W., Song B.J., Park Y.S., Lee H.B. (2015). Enhancement parameters on dynamic contrast enhanced breast MRI: Do they correlate with prognostic factors and subtypes of breast cancers?. Magn. Reson. Imaging.

[8] Teixeira S.R.C., Camargo Júnior H.S.A., Cabello C. (2020). Background parenchymal enhancement: Behavior during neoadjuvant chemotherapy for breast cancer and relationship with a pathological complete response.. Radiol. Bras..

[9] van der Velden B.H.M., Elias S.G., Bismeijer T., Loo C.E., Viergever M.A., Wessels L.F.A., Gilhuijs K.G.A. (2017). Complementary value of contralateral parenchymal enhancement on DCE-MRI to prognostic models and molecular assays in high-risk ER^+^/HER2^−^ breast cancer.. Clin. Cancer Res..

[10] Li J., Mo Y., He B., Gao Q., Luo C., Peng C., Zhao W., Ma Y., Yang Y. (2019). Association between MRI background parenchymal enhancement and lymphovascular invasion and estrogen receptor status in invasive breast cancer.. Br. J. Radiol..

[11] Rella R., Contegiacomo A., Bufi E., Mercogliano S., Belli P., Manfredi R. (2021). Background parenchymal enhancement and breast cancer: A review of the emerging evidences about its potential use as imaging biomarker.. Br. J. Radiol..

[12] Ha R., Mango V., Al-Khalili R., Mema E., Friedlander L., Desperito E., Wynn R.T. (2018). Evaluation of association between degree of background parenchymal enhancement on MRI and breast cancer subtype.. Clin. Imaging.

[13] Mercado C.L. (2014). BI-RADS update.. Radiol. Clin. North Am..

[14] Mann R.M., Pinker K. (2019). Is background parenchymal enhancement an important risk factor for breast cancer development in women with increased risk?. Radiology.

[15] Mema E., Schnabel F., Chun J., Kaplowitz E., Price A., Goodgal J., Moy L. (2020). The relationship of breast density in mammography and magnetic resonance imaging in women with triple negative breast cancer.. Eur. J. Radiol..

[16] Ha R., Chang P., Mema E., Mutasa S., Karcich J., Wynn R.T., Liu M.Z., Jambawalikar S. (2019). Fully automated convolutional neural network method for quantification of breast MRI fibroglandular tissue and background parenchymal enhancement.. J. Digit. Imaging.

[17] Dilorenzo G., Telegrafo M., La Forgia D., Stabile Ianora A.A., Moschetta M. (2019). Breast MRI background parenchymal enhancement as an imaging bridge to molecular cancer sub-type.. Eur. J. Radiol..

[18] Liao GJ, Henze Bancroft LC, Strigel RM, Chitalia RD, Kontos D, Moy L (2020). Background parenchymal enhancement on breast MRI: A comprehensive review.. J. Magn. Reson. Imaging.

[19] Telegrafo M., Rella L., Stabile Ianora A.A., Angelelli G., Moschetta M. (2016). Breast MRI background parenchymal enhancement (BPE) correlates with the risk of breast cancer.. Magn. Reson. Imaging.

[20] Öztürk M., Polat A.V., Süllü Y., Tomak L., Polat A.K. (2017). Background parenchymal enhancement and fibroglandular tissue proportion on breast MRI: Correlation with hormone receptor expression and molecular subtypes of breast cancer.. J. Breast Health.

[21] Murakami W., Mortazavi S., Yu T., Kathuria-Prakash N., Yan R., Fischer C., McCann K.E., Lee-Felker S., Sung K. (2024). Clinical significance of background parenchymal enhancement in breast cancer risk stratification.. J. Magn. Reson. Imaging.

[22] Arefan D., Zuley M.L., Berg W.A., Yang L., Sumkin J.H., Wu S. (2024). Assessment of background parenchymal enhancement at dynamic contrast-enhanced MRI in predicting breast cancer recurrence risk.. Radiology.

